# Electrochemical Assessment of the Interfacial Response of Unfractionated Bovine Heparin by Post-Processed Chronoamperometry

**DOI:** 10.1021/acsomega.6c07879

**Published:** 2026-10-05

**Authors:** Gabriel Ferreira Coelho, Leonardo Paes Cinelli, Nelilma Correia Romeiro, Rodrigo de Siqueira Melo

**Affiliations:** † Electroanalytics and Advanced Materials Group, Multidisciplinary Institute of Chemistry, Multidisciplinary Center UFRJ-Macaé, 28125 Federal University of Rio de Janeiro, Macaé, Rio de Janeiro CEP: 27930-560, Brazil; ‡ Glicomedicines Group - Institute of Pharmaceutical Sciences, Multidisciplinary Center UFRJ-Macaé, Federal University of Rio de Janeiro, Macaé, Rio de Janeiro CEP: 27930-560, Brazil; § Laboratório Integrado de Computação Científica (LICC), Multidisciplinary Institute of Chemistry, Multidisciplinary Center UFRJ-Macaé, Federal University of Rio de Janeiro, Macaé, Rio de Janeiro CEP: 27930-560, Brazil

## Abstract

Unfractionated bovine heparin (UFH-bov) is a highly sulfated and polydisperse glycosaminoglycan whose interfacial behavior can influence electrochemical mass transport. In this study, chronoamperometric current–time transients obtained for UFH-bov-containing solutions were numerically integrated to generate charge–time profiles and evaluate the concentration-dependent electrochemical response. The corresponding plots of accumulated charge as a function of the square root of time exhibited approximately linear regions within selected analysis intervals. However, UFH-bov is not directly electroactive under the experimental conditions employed and does not undergo a defined stoichiometric electron-transfer reaction. Consequently, the measured charge cannot be quantitatively related to molecular UFH-bov flux through the classical Cottrell–Anson formalism, and the experimental slopes were not converted into molecular diffusion coefficients. Instead, the slope of the selected *Q*_*t*_ versus *t* ^1/2^ region is interpreted only as an operational descriptor of the UFH-bov-dependent electrochemical response. The observed concentration dependence is consistent with interfacial phenomena, including adsorption, partial electrode blocking, and modulation of background electrochemical processes. This revised interpretation provides a conservative framework for describing square-root-of-time-dependent electrochemical behavior in the presence of a non-directly electroactive sulfated polysaccharide without assigning an unsupported molecular diffusion coefficient.

## Graphical abstract

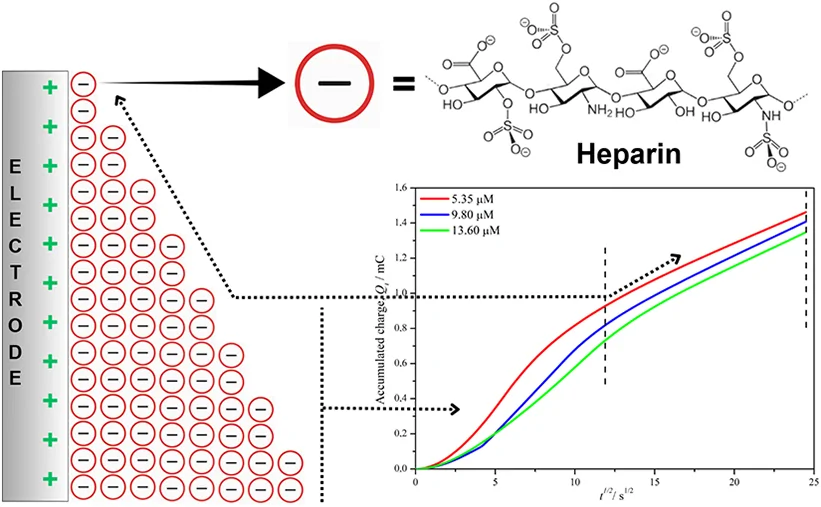

## 1 Introduction

Diffusion is a fundamental mass-transport process, but the physical meaning of a diffusion coefficient depends on the experimental framework and the transport phenomenon being considered. In multicomponent systems, self-diffusion and mutual diffusion represent conceptually distinct quantities.^1,2^ Self-diffusion describes the translational motion of a molecular species without requiring a macroscopic composition gradient, whereas mutual diffusion describes the relative transport of components associated with a concentration or composition gradient and may depend on mixture composition and non-ideal interactions. Consequently, diffusion coefficients obtained from different experimental techniques and transport regimes should not be assumed to be directly interchangeable.^1,2^ Even within a given methodological framework, numerical estimation of diffusion coefficients can be sensitive to fitting procedures and statistical treatments, as demonstrated in computer-simulation studies.^3^

In classical potential-step electrochemistry, consumption or generation of an electroactive species at the electrode-solution interface establishes a time-dependent concentration profile. Under the assumptions of semi-infinite linear diffusion and a well-defined electrode reaction, the current transient can be described by the Cottrell relationship,^4,5^ and integration of the current provides charge-time data that can be treated through the Anson formalism.^4^ Quantitative extraction of diffusion parameters from electrochemical transients requires fulfillment of the assumptions underlying the corresponding transport model.^4,6^ Electrode geometry, background currents, electrical double-layer charging, adsorption, changes in effective electroactive area, and parallel electrochemical processes may affect transient responses and consequently the parameters derived from them.^6–8^ More broadly, transport of charged species in electrically driven systems can also be strongly influenced by the surrounding ionic environment; for example, co-ion selection has been shown to modulate passive drug diffusion in electrophoretic delivery devices.^9^

For therapeutic macromolecules more generally, physicochemical attributes such as molecular weight, molecular size, ionization behavior, and solubility can influence the pharmacokinetic behavior.^10^ This distinction is particularly relevant to macromolecular polyelectrolytes. Unfractionated heparin (UFH) is a highly sulfated, strongly anionic, structurally heterogeneous, and polydisperse glycosaminoglycan. Molecular dynamics and NMR studies have demonstrated the substantial conformational flexibility of heparin,^11^ whereas electrostatic interactions, counterion effects, hydration, and ionic conditions are important determinants of UFH polyelectrolyte behavior.^12^ Related sulfated polysaccharide systems likewise illustrate that macromolecular conformation, hydrodynamic dimensions, and electrokinetic behavior depend on molecular architecture and electrolyte conditions.^13,14^ The biological source of heparin is a relevant variable. Bovine-derived heparins can exhibit structural and sulfation pattern differences relative to porcine-derived preparations.^15^

In the present study, the concentration-dependent electrochemical response of UFH-bov in aqueous buffer was investigated using chronoamperometry followed by numerical integration of current–time transients. Because UFH-bov is not directly electroactive under the experimental conditions employed and does not undergo a defined stoichiometric electron-transfer reaction, the electrochemical response is not interpreted as a direct measurement of molecular self-diffusion or mutual diffusion. Instead, the analysis focuses on the approximately linear square-root-of-time dependence observed within selected intervals of the interfacially modulated electrochemical response and on the corresponding slope *S*_*Q*_ as an operational descriptor of that response.

## 2 Results and Discussion

### 2.1 Chronoamperometric Curves

Chronoamperometric curves of UFH-bov at different concentrations are shown in Figure 1. In general, the initial current is high and decreases over time, approaching a baseline after a few minutes. Increasing the UFH-bov concentration resulted in a lower initial current and altered the subsequent current decay profile, indicating a concentration-dependent modification of the electrode-solution interface.

**Figure 1:**
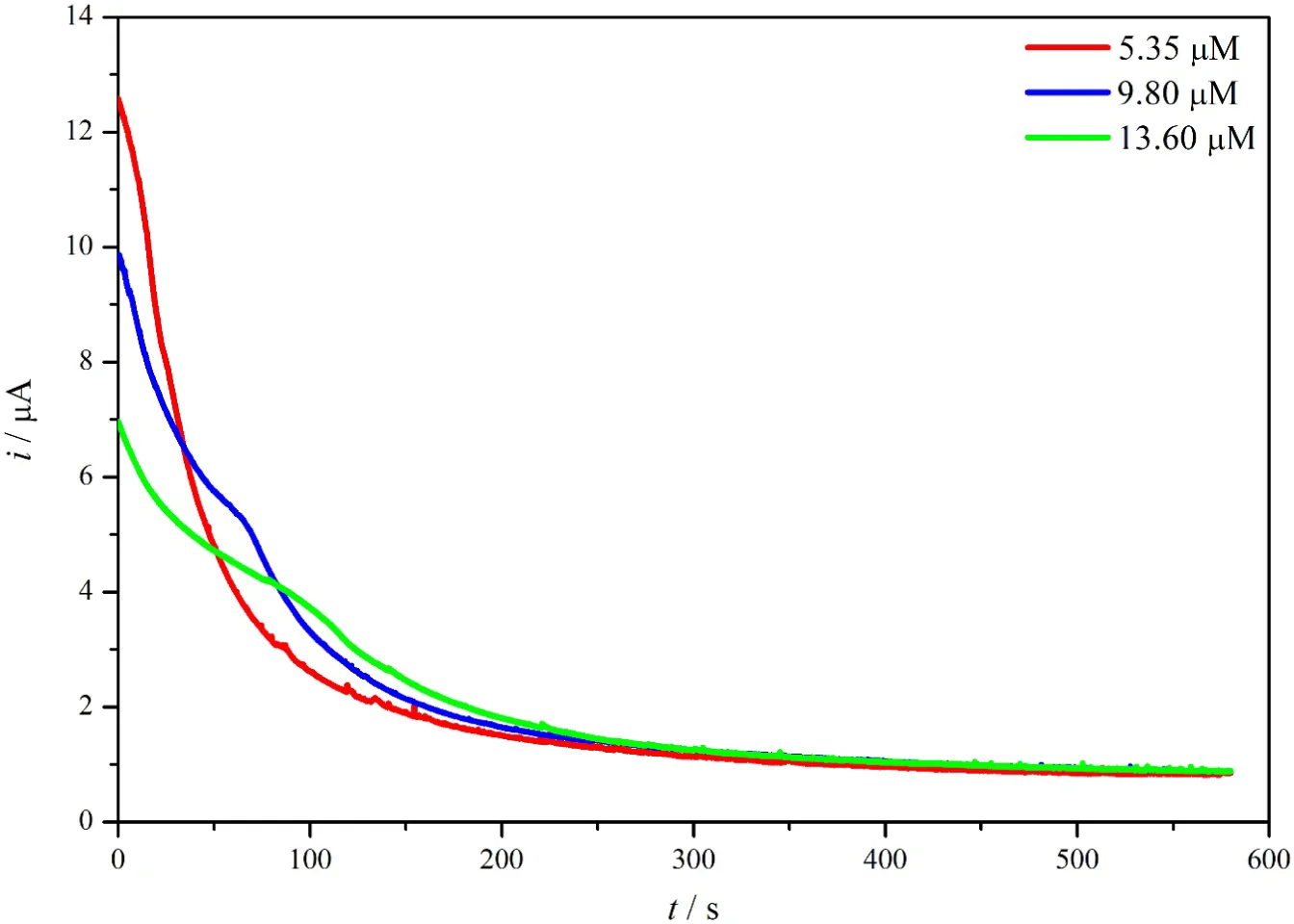
Chronoamperometric current–time responses obtained for solutions containing UFH-bov at nominal concentrations of 5.35 μM, 9.80 μM, and 13.60 μM under the experimental conditions described in Section 4.3.

UFH-bov is not intrinsically electroactive under the experimental conditions employed. Therefore, the current measured at +1.0 V is not attributed to a defined oxidation of heparin functional groups but is predominantly attributed to background electrochemical processes occurring at the glassy carbon electrode. UFH-bov modifies this response through interfacial phenomena, including adsorption or accumulation at the interface, partial electrode blocking, and modulation of the mass transport. Accordingly, the chronoamperometric response is interpreted as a probe of the UFH-bov-dependent interfacial electrochemical response rather than as direct electrolysis of UFH-bov.

The highly anionic character of UFH-bov, arising mainly from sulfate and carboxylate groups, favors strong electrostatic and hydration interactions in solution and may contribute to its interfacial behavior. Increasing UFH-bov concentration can therefore increase surface coverage and hinder access of background electroactive species or solvent to the electrode surface, consistent with the progressive decrease in the measured current. Although minor oxidative processes at the applied anodic potential cannot be completely excluded, the experimental response does not provide evidence for a defined stoichiometric redox reaction of UFH-bov.^7,12^

The investigated nominal UFH-bov concentration range was selected to provide measurable concentration-dependent changes in the electrochemical response while minimizing excessive interfacial blocking at higher concentrations.

During the initial stage of the chronoamperometric experiment, the measured current contains a substantial capacitive contribution associated with charging of the electrode–solution double layer, together with background Faradaic processes. Consequently, the early response is not interpreted using a purely diffusion-controlled Cottrell model. As time increases, the relative contribution of capacitive charging decreases and slower interfacial and mass-transport processes become increasingly important.^6,7^

Numerical integration of the chronoamperometric responses yielded the charge–time profiles shown in Figure 2. The accumulated charge increased with time and decreased with increasing UFH-bov concentration, indicating a concentration-dependent modification of the electrochemical response. This behavior is consistent with interfacial blocking and/or adsorption of the anionic polymer, which may modulate background electrochemical processes at the electrode-solution interface. The temporal dependence of the accumulated charge is further evaluated through the *Q*_*t*_ versus *t* ^1/2^ representation discussed in Section 2.2.

**Figure 2:**
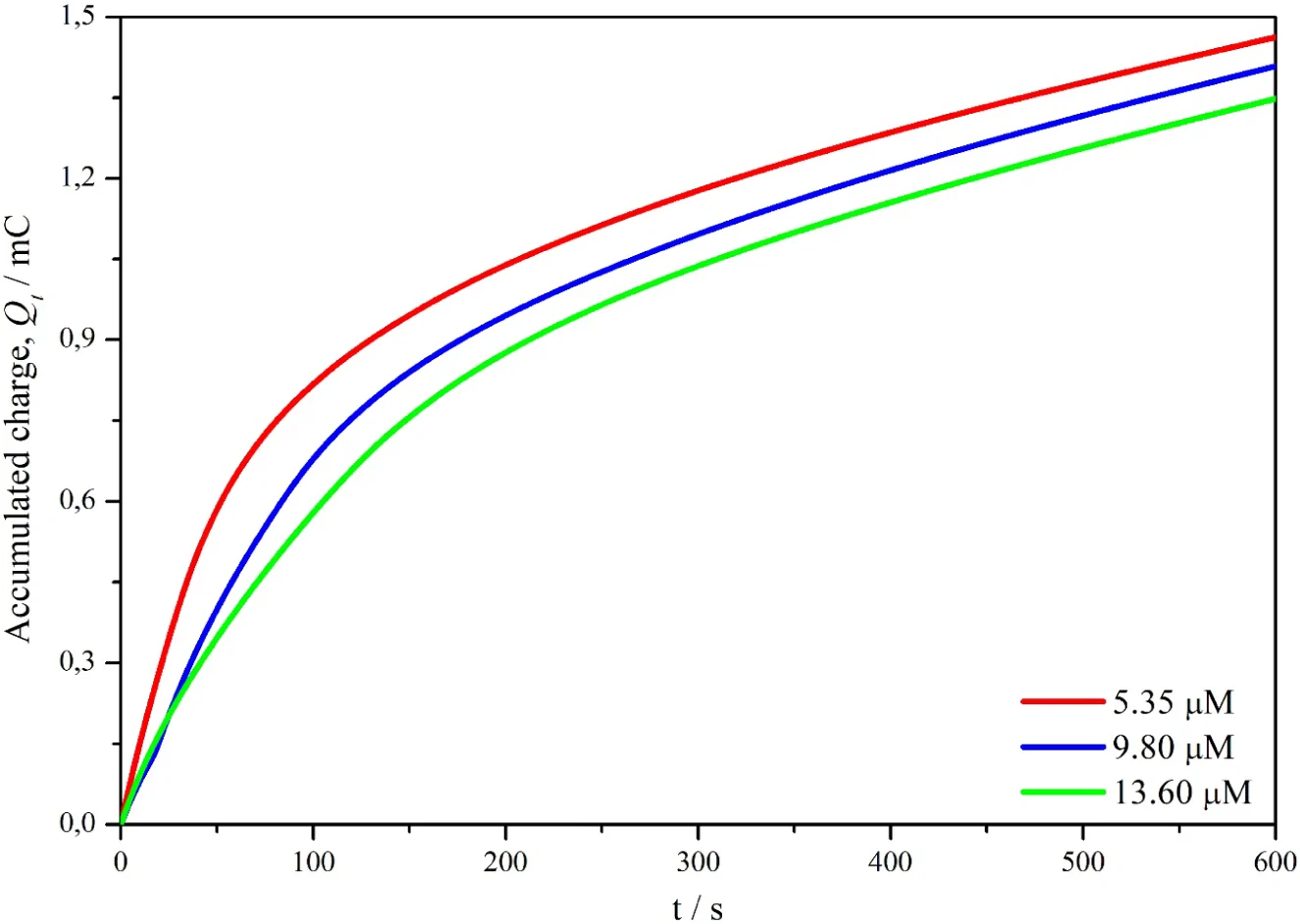
Charge–time profiles obtained by numerical integration of the chronoamperometric responses for nominal UFH-bov concentrations of 5.35, 9.80, and 13.60 μM.

### 2.2 Square-Root-of-Time Representation and Interfacial Electrochemical Analysis

Figure 3 shows accumulated charge (*Q_t_*) as a function of the square root of time (*t* ^1/2^) for the investigated nominal UFH-bov concentrations. Selected regions of the curves exhibited an approximately linear dependence on *t* ^1/2^ within the corresponding analysis intervals.

**Figure 3:**
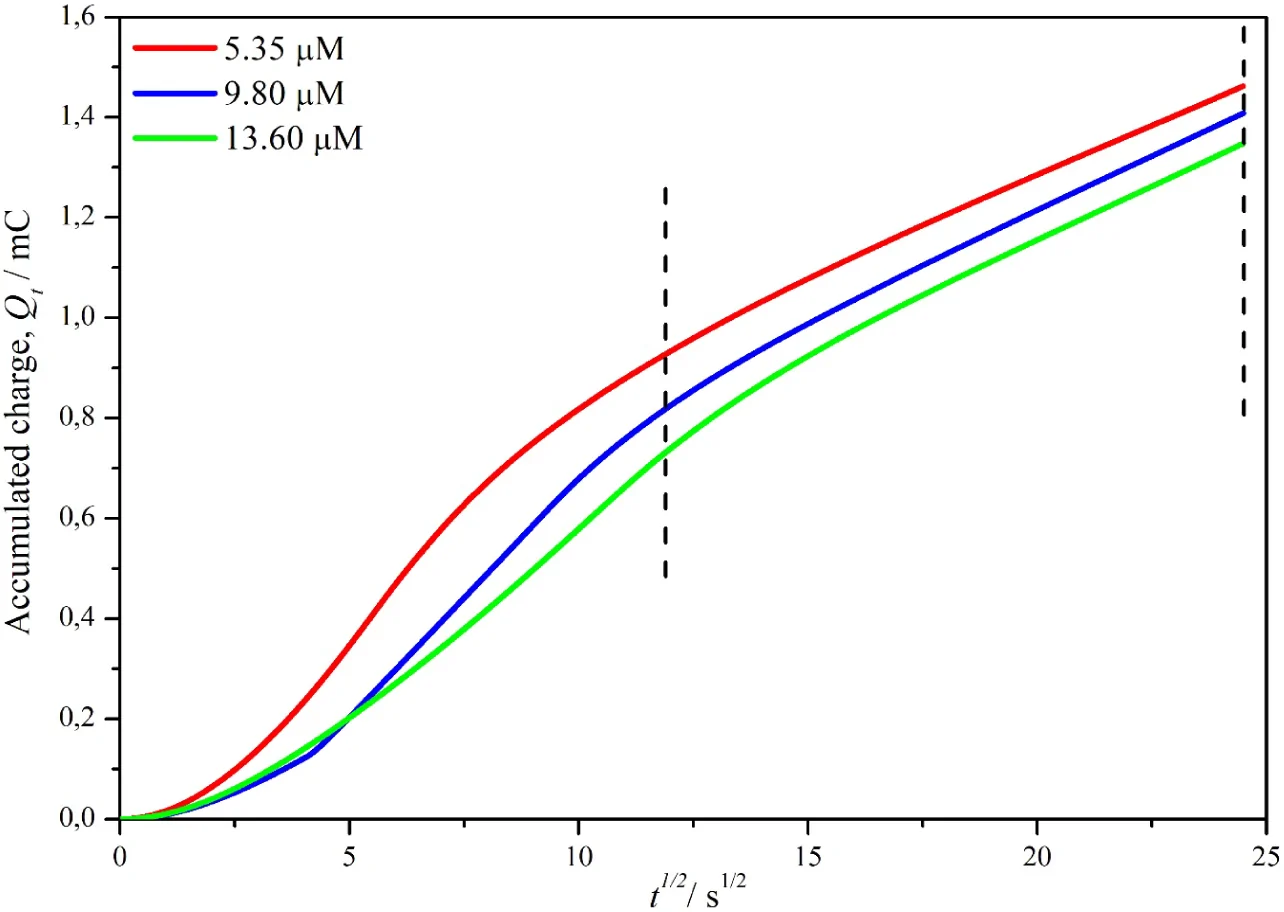
Anson-type representation of accumulated charge (*Q_t_*) as a function of the square root of time (*t* ^1/2^) for nominal UFH-bov concentrations of 5.35, 9.80, and 13.60 μM. Dashed vertical lines delimit the selected analysis interval. The approximately linear *Q_t_* versus *t* ^1/2^ dependence within this interval is used only to describe the temporal behavior of the electrochemical response and is not employed to calculate a molecular diffusion coefficient.

In the classical Cottrell-Anson framework, the slope of a *Q*_*t*_ versus *t* ^1/2^ plot depends on the stoichiometric number of electrons transferred, the electrode area, the bulk concentration of the electroactive species, and its diffusion coefficient.^4^ Quantitative extraction of a molecular diffusion coefficient therefore requires a defined electrode reaction and fulfillment of the assumptions underlying the corresponding mass-transport model.^4,5,16^ The validity of these assumptions is particularly important because background electrochemical processes, adsorption, changes in effective electroactive area, and other interfacial phenomena may influence transient electrochemical responses and the parameters derived from them.^6,8^

In the present system, UFH-bov does not undergo a defined stoichiometric electron transfer reaction under the experimental conditions employed. Consequently, an electron-transfer number cannot be independently assigned to UFH-bov, and the measured charge cannot be quantitatively equated with the molecular UFH-bov flux. For this reason, the experimental slopes were not converted to molecular diffusion coefficients.

Instead, the slope of the selected approximately linear region, denoted *S_Q_*, is retained only as an operational descriptor of the temporal electrochemical response, as defined in Section 4.4. The concentration-dependent decrease in current and accumulated charge observed in Figures 1 and 2 is consistent with increasingly pronounced interfacial effects, including adsorption or accumulation of UFH-bov at the electrode–solution interface and partial electrode blocking. The highly anionic and polyelectrolytic character of UFH may contribute to these effects through electrostatic, hydration, and surface-interaction phenomena.^7,12^

Importantly, approximately linear *Q*_*t*_ versus *t* ^1/2^ regions do not demonstrate that the overall electrochemical process is purely diffusion controlled. Electrical double-layer charging and relaxation, adsorption, partial electrode blocking, possible changes in the effective electroactive area, and background Faradaic processes may occur concurrently. Accordingly, the observed *t* ^1/2^ dependence is interpreted conservatively as an approximately linear temporal dependence within the selected analysis interval and not as validation of classical Cottrell behavior or as evidence of a unique transport mechanism.

Therefore, *S*_*Q*_ should not be interpreted as a molecular self-diffusion coefficient, a mutual-diffusion coefficient, an apparent molecular diffusion coefficient, or an electrochemical kinetic constant. Likewise, direct numerical comparison of *S*_*Q*_ with diffusion coefficients obtained by DOSY NMR, Taylor dispersion, dynamic light scattering, or related techniques is not appropriate because these methods probe physically distinct transport quantities.

## 3 Conclusions

This study investigated the concentration-dependent electrochemical response of unfractionated bovine heparin using chronoamperometry followed by numerical integration of current–time transients. Increasing UFH-bov concentration produced lower current and accumulated-charge responses, consistent with progressive modification of the electrode–solution interface.

Selected regions of the accumulated charge plots exhibited an approximately linear dependence on *t* ^1/2^ within the selected analysis interval. However, because UFH-bov is not directly electroactive and does not undergo a defined stoichiometric electron-transfer reaction, the measured charge cannot be quantitatively assigned to molecular UFH-bov flux through the classical Cottrell-Anson formalism. Consequently, the experimental slopes were not converted to absolute molecular diffusion coefficients.

The electrochemical response is instead interpreted as the combined outcome of UFH-bov-dependent interfacial phenomena, including adsorption, partial electrode blocking, modulation of background Faradaic processes, and other time-dependent interfacial contributions. This more conservative interpretation defines the scope of the present electrochemical approach and avoids assigning an unsupported molecular transport coefficient. Future studies combining electrochemical measurements with independent diffusion techniques and direct interfacial characterization will be required to establish a quantitative relationship between the observed electrochemical response and molecular UFH-BoV transport.

## 4 Experimental Section

### 4.1 Chemicals

A stock solution of 1.0 mg mL^–1^ was prepared gravimetrically by dissolving 0.100 g of UFH-bov (100 IU mg^–1^, heparin sodium, CAS registry no 9041-08-1, Adeste Corporation) in deionized water and diluting to 100 mL in a volumetric flask. The stock solution was stored at 4 °C until use. Because unfractionated heparin is a polydisperse macromolecular mixture rather than a chemically homogeneous compound with a single molecular mass, an average molecular mass of 15,000 g mol^–1^ was adopted exclusively for nominal molar-concentration conversion.^17^ Accordingly, concentration values expressed on a molar basis throughout the manuscript are referred to as nominal UFH-bov concentrations rather than absolute molecular concentrations.

Britton–Robinson (B–R) buffer was prepared using boric acid (CAS registry no 10043-35-3), acetic acid (CAS registry no 64-19-7), phosphoric acid (CAS registry no 7664-38-2), and sodium hydroxide (CAS registry no 1310-73-2). The B–R buffer solution (0.2 mol L^–1^) was adjusted to pH 5.8 by gradual addition of 1.0 mol L^–1^ NaOH under constant stirring. All solutions were prepared using deionized water (specific conductivity < 0.5 μS cm^–1^).

### 4.2 Electrochemical Measurements

Chronoamperometric experiments were carried out using a PGSTAT-128N potentiostat/galvanostat (Metrohm Autolab) equipped with a glassy carbon working electrode (geometric area = 0.03 cm^2^), a Ag/AgCl reference electrode, and a platinum auxiliary electrode. Data acquisition was performed by using NOVA 2.1 software (Metrohm Autolab).

Before each chronoamperometric measurement, the glassy carbon working electrode was mechanically polished with an aqueous alumina slurry (0.05 μm particle size) on a polishing cloth to obtain a clean and reproducible surface. After polishing, the electrode was thoroughly rinsed with deionized water to remove residual alumina particles.

Because UFH-bov can interact with the electrode–solution interface and promote adsorption and partial surface blocking, the glassy carbon electrode was regenerated between successive measurements. The regeneration procedure consisted of repolishing the electrode with 0.05 μm alumina, followed by thorough rinsing with deionized water. This procedure was used to minimize carryover from the preceding solution and to restore a comparable glassy-carbon surface before each new chronoamperometric experiment.

### 4.3 General Procedure

In a 25 mL electrochemical cell, 10.0 mL of B–R buffer solution (pH 5.8) was added, followed by the appropriate volume of the UFH-bov stock solution (1.0 mg mL^–1^). The final volume was adjusted to 15.0 mL with deionized water. UFH-bov stock-solution volumes of 1.204, 2.205, and 3.060 mL were used to obtain nominal UFH-bov concentrations of 5.35, 9.80, and 13.60 μM, respectively. These nominal molar concentrations were calculated using the average molecular mass of 15,000 g mol^–1^ defined in Section 4.1. The mixtures were homogenized before the electrochemical measurements.

The ionic environment of the B–R buffer may influence the conformation, charge distribution, hydration, and interfacial behavior of UFH-bov. Accordingly, the electrochemical response described in this study should be interpreted within the specific solution and interfacial conditions employed.^11,12^

Chronoamperometric measurements were recorded for 600 s under an applied potential of 1.0 V. This potential was selected to provide a measurable response but may also involve background electrochemical processes at the glassy carbon surface. All experiments were conducted at 25 ± 1 °C. Each experimental condition was measured in technical triplicate using the same solution-preparation condition. The curves shown in Figures 1–3 represent the pointwise mean of the three technical replicate measurements. The glassy carbon working electrode was regenerated between successive measurements according to the procedure described in Section 4.2.

The investigated UFH-bov concentration range was selected to provide measurable concentration-dependent changes in the electrochemical response while limiting excessive interfacial blocking at higher concentrations.

### 4.4 Data Treatment

Chronoamperometric current–time transients were numerically integrated according to:

$$Q_{t}\left(t\right)=\int_{0}^{t}i\left(\tau \right)d\tau$$

where *Q*_*t*_(*t*) is the accumulated charge at time *t*, *i*(τ) is the measured current at the integration time τ, and τ is the integration variable (s). When current is expressed in *A* and time in seconds, the resulting charge is obtained in C and then converted to mC for graphical representation. The charge–time profiles shown in Figure 2 were obtained by numerical integration of the original NOVA current–time datasets.

For a directly electroactive species undergoing a defined electrode reaction under semi-infinite linear diffusion, the current transient following a potential step is classically described by the Cottrell equation:^5,16^

1 $$i\left(t\right)=nFAC_{0}{\left(\frac{D}{\pi t}\right)}^{1/2}$$

where *i*(*t*) is the current at time *t* (A), *n* is the stoichiometric number of electrons transferred in the defined electrode reaction, *F* is the Faraday constant (96485 C mol^–1^), *A* is the geometric electrode area (cm^2^), *C*_0_ is the bulk concentration of the electroactive species (mol cm^–3^), *D* is the diffusion coefficient (cm^2^ s^–1^), and *t* is time (s). The quantitative application of the Cottrell relationship requires fulfillment of the assumptions underlying the corresponding transient model; when electrode or transport conditions deviate from the idealized case, modified treatments may be required.^5,6,16,17^

Integration of eq 1 with respect to time gives the classical Anson relationship for the diffusion-controlled Faradaic charge:^5^

2 $$Q_{f}\left(t\right)=2nFAC_{0}{\left(\frac{Dt}{\pi }\right)}^{1/2}$$

where is *Q*_*f*_ (*t*) is the Faradaic charge associated with the defined electroactive process.

The experimentally measured total charge may additionally contain contributions from electrical double-layer charging and interfacial adsorption.^5,7^ Thus, the total accumulated charge can be represented as:

3 $$Q_{t}=Q_{f}+Q_{dl}+Q_{ads}$$

where *Q*_*t*_ is the total accumulated charge, *Q*_*dl*_ is the contribution associated with electrical double-layer charging, and *Q*_*ads*_ represents the charge contribution associated with interfacial adsorption-related processes.

Substitution of eq 2 into eq 3 gives the classical representation:

4 $$Q_{t}\left(t\right)=2nFAC_{0}{\left(\frac{Dt}{\pi }\right)}^{1/2}+Q_{dl}+Q_{ads}$$

Under the assumptions of the classical Cottrell–Anson model, a plot of *Q*_*t*_ as a function of *t* ^1/2^ contains a linear contribution whose slope is:^16^

5 $$S_{Q}=2nFAC_{0}{\left(\frac{D}{\pi }\right)}^{1/2}$$

where *S*_*Q*_ is the slope of the selected linear region of the *Q*_*t*_ versus *t* ^1/2^ representation. Quantitative interpretation of this slope in terms of a molecular diffusion coefficient requires fulfillment of the assumptions underlying the electrochemical transport model.^4,6,16^

In the present system, however, UFH-bov is not directly electroactive under the experimental conditions employed and does not undergo a defined stoichiometric electron transfer reaction at the electrode surface. Consequently, the electron-transfer number required by eq 5 cannot be independently assigned to UFH-bov. Moreover, the measured charge cannot be assumed to be quantitatively proportional to the molecular UFH-bov flux because background Faradaic and interfacial processes contribute to the experimental response. Therefore, eq 5 is not rearranged to obtain a molecular diffusion coefficient for UFH-bov.

For the present analysis, the experimentally observed slope of the selected approximately linear region is therefore defined operationally as:

6 $$S_{Q}={\left(\frac{dQ_{t}}{dt^{1/2}}\right)}_{\mathrm{selected linear region}}$$

For the present system, *S*_*Q*_ is used exclusively as an operational descriptor of the measured electrochemical response. No molecular diffusion coefficient was calculated from *S*_*Q*_.

The approximately linear dependence observed within the selected interval is treated as an empirical square root of time dependence and does not exclude concurrent contributions from electrical double-layer charging, adsorption, partial electrode blocking, possible changes in the effective electroactive area, or background Faradaic processes. Accordingly, *S*_*Q*_ should not be interpreted as a molecular self-diffusion coefficient, a mutual-diffusion coefficient, an apparent molecular diffusion coefficient, or an electrochemical kinetic constant. The *Q*_*t*_ versus *t* ^1/2^ representations and linear regressions were generated by using OriginPro version 8.5 (OriginLab Corporation).
